# Prevalence and determinants of self-reported functional status among older adults residing in the largest refugee camp of the world

**DOI:** 10.1186/s12877-023-04067-x

**Published:** 2023-06-01

**Authors:** Afsana Anwar, Uday Narayan Yadav, Md. Nazmul Huda, M. A. Rifat, ARM Mehrab Ali, Probal Kumar Mondal, Abu Ansar Md. Rizwan, Suvasish Das Shuvo, Sabuj Kanti Mistry

**Affiliations:** 1 Social Assistance and Rehabilitation for the Physically Vulnerable (SARPV), SARPV Complex, Link Road, Cox’s Bazar, 4700 Bangladesh; 2 National Centre for Epidemiology and Population Health, The Australian National University, Canberra, ACT 2601 Australia; 3 Centre for Primary Health Care and Equity, University of New Sotuh Wales, Kensington, New South Wales 2052 Australia; 4 Translational Health Research Institute, School of Health Science, Western Sydney University, Campbelltown, New South Wales 2560 Australia; 5 Department of Global Public Health, Karolinska Institutet, Solna, Sweden; 6 ARCED Foundation, 13/1, Pallabi, Mirpur-12, Dhaka, 1216 Bangladesh; 7 Department of Nutrition and Food Technology, Jashore University of Science and Technology, Jashore, 7408 Bangladesh; 8 Department of Public Health, Daffodil International University, Dhaka, 1207 Bangladesh; 9 Brain and Mind Centre, The University of Sydney, 94 Mallet St, Camperdown, New South Wales 2050 Australia

**Keywords:** Functional status, Older adults, Rohingya, Bangladesh

## Abstract

**Background:**

The older adults of refugee camps might be vulnerable to exhibiting limited functional abilities because of the limited resources available to create a supportive environment for older population in the camps. This study aims to explore the prevalence and determinants of self-reported functional status among the older adults residing in the Rohingya refugee camp in Bangladesh.

**Methods:**

This cross-sectional study was conducted on 864 older adults aged 60 years and above living in five selected sub-camps of Rohingya refugee camp in Cox’s Bazar, Bangladesh. Data were collected through face-to-face interviews of the participants between November-December 2021. Functional status was measured using the Barthel Index. Information on participants’ sociodemographic characteristics, self-reported chronic diseases and lifestyle characteristics were also collected. A multiple logistic regression model was used to assess the factors associated with self-reported functional abilities among the participants.

**Results:**

The overall percentage of people having limited self-reported functional ability was 26.5% (male: 22.6% and female: 31.5%) with inability most found in grooming (33.2%), bathing (31.8%), stair using (13.2%) and mobility (10.7%). In the final adjusted model, having age of 80 years or more (aOR = 2.01,95% CI: 1.08,3.75), being female (aOR = 1.44, 95% CI: 1.04,2.0), having low memory or concentration (aOR = 1.83, 95% CI: 1.30,2.56), loneliness (aOR = 2.89, 95% CI:1.74,4.80) and living with aid alone (aOR = 2.89, 95% CI: 1.74,4.80) were found to be associated with self-reported limited functional ability.

**Conclusion:**

The findings of this study highlight the need for attention from policymakers and public health practitioners on addressing functional limitations among older adults residing in the Rohingya refugee camp. Our findings emphasize the need for the development of comprehensive interventions that can address the wider unmet needs (e.g., ensuring family/caregiver support, engaging in social and physical activities, providing nutritional support packages, etc.) to improve the health and well-being of older Rohingya adults.

**Supplementary Information:**

The online version contains supplementary material available at 10.1186/s12877-023-04067-x.

## Background

The world is experiencing tremendous growth in size and proportion of older adults because of the increase in life expectancy and low fertility rates [1]. The World Health Organization (WHO) projected that worldwide, the number of older adults will be increased from 1 billion in 2020 to 1.4 billion and 2.1 billion by 2030 and 2050, respectively [2]. This increase in ageing population is evidenced to be associated with increased prevalence of chronic diseases [1] and functional inabilities [3].

Rohingyas are Muslim minorities of Myanmar who faced brutal military attack in their motherland and took refuge in many Asian counties but mainly in neighbouring Bangladesh [4, 5]. In Bangladesh, they settled in Cox’s Bazar, a southern district of the country and is the largest refugee camp of the world [4]. According to the United Nations High Commission for Refugees (UNHCR), of the total 950,972 Rohingyas residing in the refugee camp of Bangladesh, 38,038 were older adults aged 60 years and above [6]. Rohingya camp is highly populated, living around 40,000 people per square kelometer [7]. The residents exhibit very poor living conditions and are cramped in their tiny allocated spaces with their families in the congested camp, which is one-fourth of the required space for a single person [8]. This poor living condition is also associated with lack of access to clean water, sanitation and hygiene (WASH) and health services [9]. There are no available tap water supply and main source of water are a few swallow-tubewells, which in-fact does not provide clean water. Furthermore, nearly twenty persons share a single outdoor latrine with long wait times for washing and bathing [7]. Due to limited facilities and crowded environment, existing WASH services (tubewells, toilets etc.) are not equitably distributed, resulting in long wait times and travels for many camp dwellers to undertake bathing and grooming. This can be a concern for older adults, particularly for those who have chronic conditions, disability and limited mobility.

The unhealthy living conditions, limited access to WASH services, challenges in accessing health and welfare services made the older adults residing in Rohingya camp physically and psychologically vulnerable [10, 11]. This could have a significant impact on their daily functional ability. Functional ability of a person can be measured in terms of the activities of daily living (ADL) [12]. ADL includes activities related to taking care of own’s body and are essential for survival and well-being, which includes bathing, dressing, toileting, transferring, and feeding [13, 14]. ADL is crucial for older adults because the inability to perform essential activities for daily living requires more medical attention and nursing care and comes with an increased risk of hospitalization [13]. Worldwide, several studies documented limited functional ability of older adults measured by ADL. A Malaysian study reported that 24.7% of older adults in rural areas had functional limitations in basic ADL items [15]. In Nigeria, 28.3% of older adults residing in rural areas had a dependency to do at least one activity of ADL [16]. While ADL represents the functional condition of an individual, its measurement and interpretation is contextual and very much dependent of the social environment at which the person lives [17–19]. Evidence demonstrates that supervised exercise or being active in older age (> 60 years) are safe and effective in preventing or attenuating functional and cognitive decline [20, 21]. Similarly, it was also found that multicomponent exercise-training programme for older adults could reverse the loss of ability to perform daily living activities (i.e., toilet use, transfers, mobility, and stair climbing) [22].

While there is limited evidence available from refugee settings, a study conducted on Syrian older refugees showed that 60% of older refugees faced difficulties in doing ADL activities [23]. A study conducted among Syrian refugees in Istanbul shows that 24.7% were physically disabled and incapable of doing functional activities [24]. It is also important to note that percentage of people with limited functional abilities can be significantly higher among the older adults from the refugee camp settings than those in the plain land. For example, a recent study conducted on rural Nepal community-dwelling older adults showed that 8.3% of participants had functional dependency [25].

There is scarce information about the functional status of the older adults residing in Rohingya refugee camp in Bangladesh. Using the World Health Organization Disability Assessment Schedule (WHO-DAS), a study found that Rohingya adult people living in Bangladeshi Rohingya camp had higher functional impairment than those living in camp of Malaysia [26]. Meanwhile, Andrew and colleagues (2017) found that 57% of the adult Rohingya population aged 18–59 years had reported mild to extreme difficulties in doing daily activities [27]. However, these studies did not focus on older population and did not comprehensively explored functional limitations. Therefore, the present study aims to examine the prevalence of self-reported functional status and its determinants among the older adults residing in Rohingya campin Bangladesh.

## Subject and methods

### Study design and participants

This study followed a cross-sectional design and was conducted between November and December 2021. Data were collected from the older adults aged 60 years and above residing in five selected sub-camps of Rohingya refugee camp where a national NGO is currently working. The sample size of 973 was calculated based on the following assumptions: 50% prevalence of functional status (unknown), a 5% margin of error, 95% level of confidence, 80% power of the test and 25% non-response rate. Finally, 864 participants consented to take part in the study of the 973 who were approached (response rate 88.8%). The participating NGO had a list of all participants aged 60 years and above residing in the selected five sub-camps, which were used as the sampling frame for the study. A simple random sampling technique was used to select the required number of participants from this sampling frame. The age of the beneficiaries was verified using the SMART card issued to each individual residing in the camp by UNHCR. The inclusion criteria included older people aged ≥ 60 years and Rohingya people residing in the refugee camp.

### Measures

#### Outcome measure

The outcome variable for the study was the level of functional status which represents the daily activities that the older people are able to perform. Functional status was measured using the Barthel Index [37] that assesses independence or dependence on ten everyday activities such as feeding, bathing, dressing, grooming, bowel and bladder control, toilet use, transfers, mobility, and stairs use. The cumulative score for Barthel Index ranges from 0 to 100; a score of > 60 was considered having a good functional ability (that indicates independence to perform everyday tasks), and ≤ 60 indicates having a limited functional ability (or greater dependence to perform everyday tasks) [25]. This scale was previously validated in Bangladesh [38] and other South Asian countries [25, 39]. We found the scale’s reliability among the participant of the present study was acceptable (Cronbach’s α = 0.91).

#### Explanatory variables

Explanatory variables of this study were selected through an extensive review of existing literature [25, 40, 41]. Explanatory variables considered in this study were age in years (categorized as 60–69, 70–79 and ≥ 80), sex (male/female), marital status (currently married/without partner), formal education (no/yes), family size (≤ 4 or > 4), family income in Bangladeshi Taka (BDT) (living on aid alone, < 5000, ≥5000), current occupation (currently employed/unemployed and retired), living arrangements (living alone or with family), memory or concentration problems (no problem/low memory or concentration), presence of non-communicable chronic conditions (no/yes), level of physical activity (regular physical activity/none or mainly sedentary), and loneliness (yes/no). Self-reported information on pre-existing non-communicable chronic conditions (e.g. arthritis, hypertension, heart diseases, stroke, hypercholesterolemia, diabetes, chronic respiratory diseases, chronic kidney disease, and cancer) was collected.

Loneliness was measured using the 3-item UCLA Loneliness scale [36]. Briefly, each item in the scale was measured with yes/no questions. The three items included: how often do you feel: (i) lack of companionship, (ii) left out, and (iii) isolated in the last two weeks. Each item in the scale was measured in terms of 3 item Likert responses: hardly ever (1 point), some of the time (2 points), and often (3 points). The participants were classified as lonely if they answered ‘some of the time’ or ‘often’ to any item [42]. The scale was previously used among Bangladeshi population [43].

### Data collection tools and techniques

A pre-tested semi-structured questionnaire developed in the Rakhine language was used to collect the information. Data were electronically recorded in the SurveyCTO mobile app (https://www.surveycto.com/) by two enumerators, who were local residents, fluent in Rakhine dialects, and had previous experience of administering health surveys in electronic platforms. Prior to data collection, the enumerators were trained extensively on the data collection tool and techniques for three days by the research team.

The English version of the questionnaire was first translated to Rakhine dialects and then back-translated to English by two staff of the participating local NGO who understand both English and Rakhine language. The Rakhine version of the tool was piloted among a small sample (n = 10) of Rohingya older adults from the selected sub-camps to refine the language in the final version. The participants approved the tool translated into Rakhine language by the research team without any corrections or modifications. Data collection was accomplished using this final tool through face-to-face interviews of the participants and each interview took around half an hour.

### Statistical analysis

The distribution of the variables was assessed through descriptive statistics. A multiple logistic regression model was performed to explore the factors associated with limited self-reported functional abilities among the participants. A backward elimination criterion with the Akaike information criterion (AIC) was employed to select the final model. Briefly, the backward elimination algorithm starts with a full model (model with all potential variables) and drops one by one variable from the model based on the statistical significance of that variable. The adjusted odds ratio, p-value, and 95% confidence interval (95% CI) for the final multiple linear regression analysis model are reported in the main table. We have also checked for any missing data, outliers and model diagnostics such as the Hosmer-Lemeshow test to assess the goodness of fit for the model, area under the receiver operating characteristic (AUROC) curve to check model’s discriminating accuracy, and the variance inflation factor (VIF) to test for multicollinearity. All analyses were performed using the statistical software package Stata (Version 17.0).

## Results

### Characteristics of the participants

The sociodemographic characteristics of the participants in this study are presented in Table 1. Majority of the participants were aged between 60 and 69 years (72.3%), male (56.3%), married (79.1%), had a family size of 4 or more people (56.9%) and were living on aid alone (67.1%). Most of the participants were living alone (90.5%), had no formal schooling (89%) and were unemployed or retired (89.1%). Around half of the participants had low memory or concentration (58%) and was suffering from non-communicable chronic conditions (50.1%). Also, the majority of the participants had a sedentary lifestyle (65.9%) and were lonley (81.1%).

**Table 1:** Characteristics of the study participants (N = 864)

Characteristics	n	%	Functional abilites (%)		
			Good (n = 635, 73.5%)	Limited (n = 229, 26.5%)	*P-value*
Age (year)					
60–69	625	72.3	76.0	24.0	0.006
70–79	190	22.0	69.5	30.5	
>= 80	49	5.7	57.1	42.9	
Sex					
Male	486	56.3	77.4	22.6	0.003
Female	378	43.8	68.5	31.5	
Marital status					
Married	683	79.1	74.8	25.2	0.087
Without partner	181	21.0	68.5	31.5	
Formal schooling					
No formal schooling	769	89.0	73.7	26.3	0.654
Having formal schooling	95	11.0	71.6	28.4	
Family size					
≤ 4	372	43.1	71.0	29.0	0.143
> 4	492	56.9	75.4	24.6	
Family monthly income (BDT)*					
Living on aid only	580	67.1	70.7	29.3	0.011
< 5000	220	25.5	77.3	22.7	
>= 5000	64	7.4	85.9	14.1	
Current occupation					
Employed	94	10.9	78.7	21.3	0.224
Unemployed/retired	770	89.1	72.9	27.1	
Living arrangement					
Living with family	782	90.5	74.3	25.7	0.099
Living alone	82	9.5	65.9	34.2	
Problem in memory or concentration					
No problem	363	42.0	80.4	19.6	< 0.001
Low memory or concentration	501	58.0	68.5	31.5	
Suffering from non-communicable chronic conditions					
No	431	49.9	71.7	28.3	0.231
Yes	433	50.1	75.3	24.7	
Level of physical activity					
Regular physical activity	295	34.1	79.7	20.3	0.003
None or mainly sedentary	569	65.9	70.3	29.7	
Feeling of loneliness					
No	163	18.9	87.7	12.3	< 0.001
Yes	701	81.1	70.2	29.8	

*1 USD ~ 100 BDT

### Functional status of the participants

Overall, around one quarter (26.5%) of the participants had limited self-reported functional ability (Table 1). The most inability to perform was observed for grooming (33.2%), followed by bathing (31.8%), stair use (13.2%), and mobility (10.7%). The distribution of the responses to each item on the Barthel index is presented in Table 2. Having limited functional abilities were significantly higher (p < 0.005) among the participants who were female, aged > = 80 years, living on aid alone, had memory or concentration problems and were lonely (Table 3).

**Table 2:** Participants’ responses to 10 items of the Barthel Index (BI) on activities of daily life

Activities	N	%
Feeding		
Inability to perform	72	8.3
Assistance required	201	23.3
Independent	591	68.4
Bathing		
Inability to perform	275	31.8
Assistance required	589	68.2
Grooming		
Inability to perform	287	33.2
Assistance required	577	66.8
Dressing		
Inability to perform	74	8.6
Assistance required	206	23.8
Independent	584	67.6
Bowel control		
Incontinent	67	7.8
Occasional accident	230	26.6
Continent	567	65.6
Bladder control		
Incontinent	69	8.0
Occasional accident	213	24.7
Continent	582	67.4
Toilet use		
Inability to perform	71	8.2
Assistance required	250	28.9
Independent	543	62.9
Transfers		
Inability to perform	75	8.7
Needs major help	54	6.3
Needs minor help	170	19.7
Independent	565	65.4
Mobility		
Inability to perform	92	10.7
Wheelchair independent	39	4.5
Walks with help of one person	187	21.6
Independent	546	63.2
Stairs		
Inability to perform	114	13.2
Assistance required	324	37.5
Independent	426	49.3

**Table 3:** Factors associated with limited functional abilities among the participants (N = 864)

Characteristics	aOR	95% CI		*P*
Age (year)				
60–69	*Ref*			
70–79	1.34	0.92	1.95	0.122
>= 80	2.01	1.08	3.75	0.027
Sex				
Male	*Ref*			
Female	1.44	1.04	2.00	0.027
Formal schooling				
No formal schooling	*Ref*			
Having formal schooling	1.55	0.92	2.59	0.096
Family monthly income (BDT)				
>= 5000	*Ref*			
< 5000	2.01	0.90	4.47	0.087
Living on aid only	2.30	1.09	4.85	0.029
Problem in memory or concentration				
No problem	*Ref*			
Low memory or concentration	1.83	1.30	2.56	< 0.001
Feeling of loneliness				
No	*Ref*			
Yes	2.89	1.74	4.80	< 0.001

### Factors associated with limited functional abilities

The factors associated with limited self-reported functional abilities are presented in Table 3. In the final model, age, sex, monthly family income, problem in memory or concentration, and feeling of loneliness were significantly associated with limited self-reported functional abilities. Participants aged ≥ 80 years were two times more likely (aOR = 2.01, 95% CI: 1.08, 3.75) to have limited self-reported functional abilities compared to the youngest age groups (60–69 years). Limited self-reported functional abilities were significantly more likely to occur among female (aOR = 1.44, 95% CI: 1.04, 2.00), participants with low memory or concentration (aOR = 1.83, 95% CI: 1.30, 2.56), and among those who felt loneliness (aOR = 2.89, 95% CI: 1.74, 4.80) compared to their respective counterparts. Also, participants who were living on aid only had 2.3 times higher odds of having limited self-reported functional abilities (aOR = 2.30, 95% CI: 1.09, 4.85) than those with a monthly family income of BDT ≥ 5000. The goodness of fit for the model was appropriate as indicated by the Hosmer-Lemeshow test (p = 0.355). The model had moderate discrimination accuracy measured through AUROC curve (Supplementary file 1) and had no serious multicollinearity with final variables (VIF < 5).

## Discussion and implications

The present study is the first to examine the functional status of older adults residing in Rohingya camp in Bangladesh. The study found that 26.5% of the older adults had limited functional ability. While no previous study explored functional limitation among older adults residing in the Rohingya refugee camp, a study reported higher limited functional impairment among adult population of Rohingya refugee camp in Bangladesh compared to that of Malaysia [26]. Meanwhile, similar to the findings of the current study, a study conducted among older Syrian and Palestinian refugees living in Lebanon reported that 10% of the participants had moderate impaired functional status and 18% had severe impaired functional status [44]. It is evident that changes in physical, mental, and psychosocial status, together with different disease conditions in old age deteriorate the functional status of the older adults [45–47]. Several factors, in particular, can limit functional abilities of the people residing in the refugee camp including man-made and natural disasters increased diseases, malnutrition, living in overcrowded settlements, poverty, poor WASH facilities, and poor access to health care [48, 49]. All these prevalent factors in the camp environment might also resulted in limited functional abilities among the Rohingya older adults in Bangladesh. Difficulties in ADL indicates lack of health and social care services. Government of Bangladesh and other development partners need to put more focus on providing basic health and social care services for older adults residing in the Rohingya refugee camp of Bangladesh.

We found that female participants were more prone to have limited functional abilities than their male counterparts in the present study is supported by previous studies from other settings of the world [54–56]. Higher prevalence of chronic conditions, and living longer than males [57] can be attributed to higher prevalence of limited functional abilities among older females than males. Studies report that females are more adapted socially to accept sickness and physical discomfort than males [54] mainly due to their financial dependence on males and gendered social norms [58]. Also, use of open latrine, particularly in night has been limited among females in Rohingya refugee camp [59]. This might have also resulted in limited functional ability among the older Rohingya females. Evidence suggests that females are generally more vulnerable [57, 60], and factors associated with their vulnerability, social conditions, contextualization, and female-friendly initiatives must be considered while designing interventions to address their limited functional abilities.

The present study also found that participants who were living on financial aid alone tended to have limited functional capacity compared to those who have some extra household income besides the financial aid. A previous study [61] also documented that poor income was associated with an increased risk of losing mobility. Increased income is associated with increased access to ambulation aid, lifelong health practices, and medical care, which increases the likelihood to maintaining good mobility in later life [61]. Inadequate income restricts the purchasing and utilization capacity of the older adults and impacts on their good dietary practices, resulting in limited functional abilities [61, 62]. In particular, having additional household income could have allowed participants to ensure having sanitary facilities i.e., soaps at home which is a great concern in camp [9] and could have had the opportunity to hire someone to help. Therefore, having a higher family income can ensure dietary diversity, contributing to higher functional abilities among the older population. Relevant authorities should look for introducing some household based income generating activities in the camp to increase household income of the people residing in the camp.

We also found that participants who were living alone and who felt loneliness had a higher chance of having limited self-reported functional ability than those who lived with families and did not feel alone. In the Rohingya community living in Bangladesh, older adults living with family generally get assistance to do activities from their family members. Their family members help them to perform their activities. Living alone or not with family members limits their opportunities to get family assistance, especially for collecting water or accessing sanitation facilities, which might increase their likelihood of having less functional abilities. There are mixed findings regarding this aspect. Some studies found that living alone or being lonely increases the chance of having limited functional ability among older adults [63, 64] because loneliness or living alone comes with having limited formal care [65]. Other studies, however, noted that loneliness or living alone is not related to increased functional problems [57, 66, 67] as higher independence is related to higher financial capacity [57]. Our study also found that participants with low memory or concentration had limited self-reported functional abilities. Prior research also pointed that memory and concentration work as predictors of older people’s functional abilities [68]. Increased functional inability with low memory or concentration is considered one of the earliest symptoms of cognitive impairment due to Alzheimer’s disease, which limit the functional capacity of older adults [25, 69].

### Implications for policy and practice

Exploring the functional status and associated factors among the older adults of the Rohingya refugee camp is especially critical as the findings may help identify the critical initiatives for successful program implementation. Our study’s findings highlight the necessity of working together with relevant stakeholders, partners, and agencies to improve health and social wellbeing for older adults in the refugee setting. Relevant authorities and stakeholders should design and implement initiatives, taking into consideration of the specific needs of older adults. Currently, very few organizations are working for older adults in the Rohingya camp. Young Power in Social Action (YPSA) and Help Age International-Bangladesh are working with older adults by offering them medical assistance, recreational activities, income-generating activities, counselling, age-friendly space initiatives, home-based care services, access to age-friendly latrines in a limited numbers of camps in small-scale [11, 70]. Handicap International, Centre For Disability in Development (CDD), Cristian Blind Mission (CBM), Social Assistance and Rehabilitation for the Physically Vulnerable (SARPV), and the International Organization for Migration (IOM) work with different types of disabilities covering all age groups. They work on providing assistive devices, physiotherapy, occupational therapy, home-based care, caregiver training, and awareness sessions [71, 72]. Organizations working with older adults in Rohingya camp need to come together to offer effective services to address functional limitations among the older population residing in the camp. Such services may consider physical exercise, psychological exercise, social engagement, counselling, income-generating activities, and nutritional intervention for target groups [73]. As programs and services for older adults are limited in Rohingya camp, donor agencies and implementing partners must consider this critical issues.

### Strengths and limitations

The present study has its own strengths and limitations. Being unique is the first strength of the study. No other study conducted in Rohingya camp in Bangladesh focused on exploring functional status and associated factors of older Rohingya adults. The study population and study area are exclusive because Rohingya camp in Bangladesh is the largest refugee settlement of the world. The study findings will add to the available literature on functional status among displaced, migrants, and refugee people. The study has its own limitations also. The study is cross-sectional, giving a snapshot of the issue, but causality has not been established. Due to restrictions on data collection capacity, a limited number of sites were selected. We use purposive sampling for selecting sub-camps that could affect the generalizability of findings. Moreover, as we reported the self-reported functional status, this could include recall bias. Nevertheless, we believe that recall bias was minimal as the data collection was assisted by local volunteers who asked the questions regarding the activities they regularly do. The possibility of reverse causality is also possible given that the bidirectional relationship between functional status and health conditions. Measurements of ADL highly depends on social settings. For example, in this case, the feeling of isolation might also be mediated by COVID-19 pandemic and lockdown in the camp, inability to bathing and using stairs might be due to poor WASH facility and infrastructures in the camp; therefore, these factors should be taken into consideration while interpreting the findings. Furthermore, this study was limited to quantitative data collection. However, it did not collect qualitative data on how ADLs were measured, nor did it explore the social conditions in which Rohingya refugees live in the camp. This highlights the necessity for further studies with mixed-method design.

## Conclusion

The findings of the present study highlights the necessity of focusing on this vulnerable population by designing and implementing interventions that can address the identified modifiable factors (i.e., economic status, loneliness) associated with limited functional abilities among older adults living in Rohingya camp of Bangladesh. Further, we suggest the need of qualitative studies using the socio-ecological approach in order to understand the underlying reasons for poor functional status that may guide the development of a holistic intervention to address the needs of older adults living in Rohingya camp. We have been sharing our findings with local policymakers and other potential stakeholders through our informal and formal networks. We are also planning to organize a dissemination seminar with non-government and governmental organizations that could use these findings to get it absorbed in policy and practice. Further, we are seeking some funding to share our findings in plain summary to study participants.

## Electronic supplementary material

Below is the link to the electronic supplementary material.

Supplementary file 1: Area under the receiver operating characteristic (AUROC) curve

## Data Availability

The datasets generated and/or analyzed during the current study are not publicly available due the organizational policy of the institution undertaking the research but are available from the corresponding author on reasonable request.

## References

[1] Brown GC (2015). Living too long: the current focus of medical research on increasing the quantity, rather than the quality, of life is damaging our health and harming the economy. EMBO Rep.

[2] Ageing. and Health [https://www.who.int/news-room/fact-sheets/detail/ageing-and-health].

[3] Bleijenberg N, Zuithoff NPA, Smith AK, De Wit NJ, Schuurmans MJ (2017). Disability in the individual ADL, IADL, and mobility among older adults: a prospective cohort study. J Nutr Health Aging.

[4] Myanmar Rohingya. : What you need to know about the crisis [https://www.bbc.com/news/world-asia-41566561].

[5] Prasse-Freeman E (2017). The rohingya crisis. Anthropol Today.

[6] Joint Government of Bangladesh - UNHCR. Population Factsheet [file:///C:/Users/smis7723/Downloads/GoB%20UNHCR%20Population%20Factsheet%20-%20Nov%20%202022.pdf].

[7] Kamal A-H, Huda M, Dell C, Hossain S, Ahmed S. Translational strategies to control and prevent spread of COVID-19 in the Rohiynga refugee camps in Bangladesh. Global Biosecur 2020, 2(1).

[8] Hossain MA, Huda MN, Ullah AKMA, Renzaho A (2022). Risk factors, contemporary challenges and psychological well-being of the Rohingya refugees in Bangladesh: policy implications. Int J Health Plann Manag.

[9] Faruque ASG, Alam B, Nahar B, Parvin I, Barman AK, Khan SH, Hossain MN, Widiati Y, Hasan ASMM, Kim M (2022). Water, Sanitation, and Hygiene (WASH) Practices and Outreach Services in settlements for Rohingya Population in Cox’s Bazar, Bangladesh, 2018–2021. Int J Environ Res Public Health.

[10] Islam MM, Nuzhath T. Health risks of Rohingya refugee population in Bangladesh: a call for global attention. J global health 2018, 8(2). 10.7189/jogh.08.020309 PMC6220352 30410735

[11] Khan HTA, Rahman MA, Molla MH, Shahjahan M, Abdullah RB. Humanitarian emergencies of Rohingya older people in Bangladesh: a qualitative study on hopes and reality. Ageing Int 2020:1–18.

[12] Miller ME, Rejeski WJ, Reboussin BA, Ten Have TR, Ettinger WH (2000). Physical activity, functional limitations, and disability in older adults. J Am Geriatr Soc.

[13] Finlayson M, Mallinson T, Barbosa VM (2005). Activities of daily living (ADL) and instrumental activities of daily living (IADL) items were stable over time in a longitudinal study on aging. J Clin Epidemiol.

[14] Tas Ü, Verhagen AP, Bierma-Zeinstra SMA, Odding E, Koes BW (2007). Prognostic factors of disability in older people: a systematic review. Br J Gen Pract.

[15] Hairi NN, Bulgiba A, Cumming RG, Naganathan V, Mudla I (2010). Prevalence and correlates of physical disability and functional limitation among community dwelling older people in rural Malaysia, a middle income country. BMC Public Health.

[16] Abdulraheem IS, Oladipo AR, Amodu MO. Prevalence and correlates of physical disability and functional limitation among elderly rural population in Nigeria. *Journal of aging research* 2011, 2011. 10.4061/2011/369894 PMC3124875 21748005

[17] Wiener JM, Hanley RJ, Clark R, Van Nostrand JF (1990). Measuring the activities of daily living: comparisons across national surveys. J Gerontol.

[18] Mlinac ME, Feng MC (2016). Assessment of activities of daily living, self-care, and independence. Arch Clin Neuropsychol.

[19] Edemekong PF, Bomgaars DL, Sukumaran S, Schoo C. Activities of Daily Living. 2022 Jul 3. *StatPearls [Internet] Treasure Island (FL): StatPearls Publishing* 2022. 29261878

[20] Izquierdo M, Duque G, Morley JE (2021). Physical activity guidelines for older people: knowledge gaps and future directions. The Lancet Healthy Longevity.

[21] Saez de Asteasu ML, Martínez-Velilla N, Zambom‐Ferraresi F, Casas‐Herrero Á, Cadore EL, Ramirez‐Velez R, Izquierdo M (2019). Inter‐individual variability in response to exercise intervention or usual care in hospitalized older adults. J cachexia sarcopenia muscle.

[22] Martínez-Velilla N, Sáez de Asteasu ML, Ramírez-Vélez R, Zambom-Ferraresi F, García-Hermoso A, Izquierdo M (2021). Recovery of the decline in activities of daily living after hospitalization through an individualized exercise program: secondary analysis of a randomized clinical trial. The Journals of Gerontology: Series A.

[23] Skinner M. The impact of displacement on disabled, injured and older syrian refugees. Forced Migration Review 2014(47):39.

[24] Polack S, Scherer N, Yonso H, Volkan S, Pivato I, Shaikhani A, Boggs D, Beck AH, Atijosan-Ayodele O, Deniz G (2021). Disability among syrian refugees living in Sultanbeyli, Istanbul: results from a population-based survey. PLoS ONE.

[25] Ghimire S, Paudel G, Mistry SK, Parvez M, Rayamajhee B, Paudel P, Tamang MK, Yadav UN (2021). Functional status and its associated factors among community-dwelling older adults in rural Nepal: findings from a cross-sectional study. BMC Geriatr.

[26] Khan S, Haque S (2021). Trauma, mental health, and everyday functioning among Rohingya refugee people living in short-and long-term resettlements. Soc Psychiatry Psychiatr Epidemiol.

[27] Riley A, Varner A, Ventevogel P, Taimur Hasan MM, Welton-Mitchell C (2017). Daily stressors, trauma exposure, and mental health among stateless rohingya refugees in Bangladesh. Transcult Psychiatry.

[28] Chakrabarty D, Mandal PK, Manna N, Mallik S, Ghosh P, Chatterjee C, Sardar JC, Sau M, Roy AKS. Functional disability and associated chronic conditions among geriatric populations in a rural community of India. Ghana Med J 2010, 44(4). 10.4314/gmj.v44i4.68913 PMC3052829 21416049

[29] Díaz-Venegas C, Reistetter TA, Wang C-Y, Wong R (2016). The progression of disability among older adults in Mexico. Disabil Rehabil.

[30] Miner SM, Liebel D, Wilde MH, Carroll JK, Zicari E, Chalupa S (2017). Meeting the needs of older adult refugee populations with home health services. J Transcult Nurs.

[31] Liu K, Coughlin T, McBride T. Predicting nursing-home admission and length of stay: a duration analysis. Med Care 1991:125–41. 10.1097/00005650-199102000-00005 1994146

[32] Burman J, Sembiah S, Dasgupta A, Paul B, Pawar N, Roy A (2019). Assessment of poor functional status and its predictors among the elderly in a rural area of West Bengal. J mid-life health.

[33] Operation Data Portal. : Refugee Situations [https://data2.unhcr.org/en/situations/myanmar_refugees ].

[34] Global strategy and action plan on ageing. and health [https://www.who.int/publications-detail-redirect/9789241513500 ].

[35] Age, Friendly. world [https://extranet.who.int/agefriendlyworld/ ].

[36] Abualghaib O, Groce N, Simeu N, Carew MT, Mont D (2019). Making visible the invisible: why disability-disaggregated data is vital to “leave no-one behind. Sustainability.

[37] Mahoney FI (1965). Functional evaluation: the Barthel index. Maryland State Med J.

[38] Naher L, Hiramoni FA, Alam N, Ahmed O (2022). Psychometric assessment of the Bangla version of the Bergen Social Media Addiction Scale. Heliyon.

[39] Mustafa K, Waqas S, Dar RK, Dar RK, Sherazi QUA, Tariq M, Asim HM (2022). Translation and validation of Barthel Index in Urdu Language for Stroke Patients. Pakistan J Med Health Sci.

[40] Mistry SK, Ali ARMM, Yadav UN, Huda MN, Ghimire S, Bestman A, Hossain MB, Reza S, Qasim R, Harris MF (2021). Difficulties faced by older Rohingya (forcibly displaced Myanmar nationals) adults in accessing medical services amid the COVID-19 pandemic in Bangladesh. BMJ global health.

[41] Tareque MI, Tiedt AD, Islam TM, Begum S, Saito Y (2017). Gender differences in functional disability and self-care among seniors in Bangladesh. BMC Geriatr.

[42] Perissinotto CM, Cenzer IS, Covinsky KE (2012). Loneliness in older persons: a predictor of functional decline and death. Arch Intern Med.

[43] Mistry SK, Ali ARMM, Yadav UN, Huda MN, Ghimire S, Saha M, Sarwar S, Harris MF (2022). Loneliness and its correlates among bangladeshi older adults during the COVID-19 pandemic. Sci Rep.

[44] Strong J, Varady C, Chahda N, Doocy S, Burnham G (2015). Health status and health needs of older refugees from Syria in Lebanon. Confl health.

[45] WHO. : World report on ageing and health. In. Geneva: World Health Organization; 2015.

[46] Fiest KM, Jette N, Roberts JI, Maxwell CJ, Smith EE, Black SE, Blaikie L, Cohen A, Day L, Holroyd-Leduc J (2016). The prevalence and incidence of dementia: a systematic review and meta-analysis. Can J Neurol Sci.

[47] van der Vorst A, Zijlstra GAR, Witte ND, Duppen D, Stuck AE, Kempen GIJM, Schols JMGA, Consortium DS (2016). Limitations in activities of daily living in community-dwelling people aged 75 and over: a systematic literature review of risk and protective factors. PLoS ONE.

[48] Enn ENN (2015). Insight into experiences of older syrian refugees in Lebanon. Field Exch.

[49] Owoaje ET, Uchendu OC, Ajayi TO, Cadmus EO (2016). A review of the health problems of the internally displaced persons in Africa. Nigerian Postgrad Med J.

[50] Wahrendorf M, Reinhardt JD, Siegrist J (2013). Relationships of disability with age among adults aged 50 to 85: evidence from the United States, England and continental europe. PLoS ONE.

[51] Connolly D, Garvey J, McKee G (2017). Factors associated with ADL/IADL disability in community dwelling older adults in the irish longitudinal study on ageing (TILDA). Disabil Rehabil.

[52] Reynolds SL, Silverstein M (2003). Observing the onset of disability in older adults. Soc Sci Med.

[53] Grönstedt H, Hellström K, Bergland A, Helbostad JL, Puggaard L, Andresen M, Granbo R, Frändin K (2011). Functional level, physical activity and wellbeing in nursing home residents in three nordic countries. Aging Clin Exp Res.

[54] Zeki Al Hazzouri A, Mehio Sibai A, Chaaya M, Mahfoud Z, Yount KM (2011). Gender differences in physical disability among older adults in underprivileged communities in Lebanon. J Aging Health.

[55] Fusco O, Ferrini A, Santoro M, Lo Monaco MR, Gambassi G, Cesari M (2012). Physical function and perceived quality of life in older persons. Aging Clin Exp Res.

[56] Cabral JF, Silva AMCd, Mattos IE, Neves ÁdQ, Luz LL, Ferreira DB, Santiago LM (2019). Carmo CNd: vulnerability and associated factors among older people using the family health strategy. Ciência & Saúde Coletiva.

[57] Seonho KIM, Myoungsuk KIM, Dallong HAN (2020). Incidence rates of disability and its associated factors among korean community-dwelling older adults. Iran J Public Health.

[58] Huda M, Hossain SZ, Dune TM, Amanullah ASM, Renzaho A (2022). The involvement of bangladeshi girls and women in sex work: sex trafficking, Victimhood, and Agency. Int J Environ Res Public Health.

[59] Lights and Safe. Access to WASH Facilities in Camps [https://reliefweb.int/report/bangladesh/lights-and-safe-access-wash-facilities-camps].

[60] Zunzunegui M-V, Alvarado B-E, Béland F, Vissandjee B (2009). Explaining health differences between men and women in later life: a cross-city comparison in Latin America and the Caribbean. Soc Sci Med.

[61] Guralnik JM, LaCroix AZ, Abbott RD, Berkman LF, Satterfield S, Evans DA, Wallace RB (1993). Maintaining mobility in late life. I. demographic characteristics and chronic conditions. Am J Epidemiol.

[62] Liu H, Wang M (2022). Socioeconomic status and ADL disability of the older adults: cumulative health effects, social outcomes and impact mechanisms. PLoS ONE.

[63] Rist PM, Liu SY, Glymour MM (2016). Families and disability onset: are spousal resources less important for individuals at high risk of dementia?. Am J Geriatric Psychiatry.

[64] Michael YL, Berkman LF, Colditz GA, Kawachi I (2001). Living arrangements, social integration, and change in functional health status. Am J Epidemiol.

[65] Asim M, Aftab M, Bhatti SH, Ahmad T, Ali Shah SM, Akram M (2021). Identifying factors causing decline in physical functionality of geriatric population. Eur J Inflamm.

[66] Sarwari AR, Fredman L, Langenberg P, Magaziner J (1998). Prospective study on the relation between living arrangement and change in functional health status of elderly women. Am J Epidemiol.

[67] Wang D, Zheng J, Kurosawa M, Inaba Y, Kato N (2009). Changes in activities of daily living (ADL) among elderly chinese by marital status, living arrangement, and availability of healthcare over a 3-year period. Environ Health Prev Med.

[68] Freilich BM, Hyer LA (2007). Relation of the repeatable battery for Assessment of Neuropsychological Status to measures of daily functioning in dementia. Psychol Rep.

[69] Tabira T, Hotta M, Murata M, Yoshiura K, Han G, Ishikawa T, Koyama A, Ogawa N, Maruta M, Ikeda Y (2020). Age-related changes in instrumental and basic activities of daily living impairment in older adults with very mild Alzheimer’s disease. Dement Geriatric Cogn Disorders Extra.

[70] Myanmar crisis [https://www.helpage.org/what-we-do/emergencies/humanitarian-response/rohingya-crisis/].

[71] Inclusive Humanitarian Response. : Access to rehabilitation services and disability mainstreaming for Rohingya crisis [https://cdd.org.bd/2022/03/23/inclusive-humanitarian-response-access-to-rehabilitation-services-and-disability-mainstreaming-for-the-rohingya-crisis/ ].

[72] Humanity. & Inclusion in Bangladesh [https://www.hi-us.org/bangladesh].

[73] Daniels R, van Rossum E, de Witte L, Kempen GIJM, van den Heuvel W (2008). Interventions to prevent disability in frail community-dwelling elderly: a systematic review. BMC Health Serv Res.

