# Supplementary material for: Prevalence and determinants of self-reported functional status among older adults residing in the largest refugee camp of the world
Source: BMC Geriatr. 2023 Jun 1;23:345. doi: 10.1186/s12877-023-04067-x (PMC10234234; doi:10.1186/s12877-023-04067-x)
Supplement: Supplementary file 1 — Area under the receiver operating characteristic (AUROC) curve [file 12877_2023_4067_MOESM1_ESM.docx]

Supplementary file 1: Area under the receiver operating characteristic (AUROC) curve
